# Selective Depletion of Eosinophils or Neutrophils in Mice Impacts the Efficiency of Apoptotic Cell Clearance in the Thymus

**DOI:** 10.1371/journal.pone.0011439

**Published:** 2010-07-06

**Authors:** Hye-Jung Kim, Eric S. Alonzo, Guillaume Dorothee, Jeffrey W. Pollard, Derek B. Sant'Angelo

**Affiliations:** 1 Immunology Program, Sloan-Kettering Institute, Memorial Sloan-Kettering Cancer Center, New York, New York, United States of America; 2 Louis V. Gerstner Jr. Graduate School of Biomedical Sciences, Memorial Sloan-Kettering Cancer Center, New York, New York, United States of America; 3 Weill Graduate School of Medical Sciences of Cornell University, New York, New York, United States of America; 4 Department of Development and Molecular Biology, Albert Einstein College of Medicine, Bronx, New York, New York, United States of America; New York University, United States of America

## Abstract

Developing thymocytes undergo a rigorous selection process to ensure that the mature T cell population expresses a T cell receptor (TCR) repertoire that can functionally interact with major histocompatibility complexes (MHC). Over 90% of thymocytes fail this selection process and die. A small number of macrophages within the thymus are responsible for clearing the large number of dying thymocytes that must be continuously cleared. We studied the capacity of thymic macrophages to clear apoptotic cells under acute circumstances. This was done by synchronously inducing cell death in the thymus and then monitoring the clearance of apoptotic thymocytes. Interestingly, acute cell death was shown to recruit large numbers of CD11b^+^ cells into the thymus. In the absence of a minor CSF-1 dependent population of macrophages, the recruitment of these CD11b^+^ cells into the thymus was greatly reduced and the clearance of apoptotic cells was disrupted. To assess a possible role for the CD11b^+^ cells in the clearance of apoptotic cells, we analyzed mice deficient for eosinophils and mice with defective trafficking of neutrophils. Failure to attract either eosinophils or neutrophils to the thymus resulted in the impaired clearance of apoptotic cells. These results suggested that there is crosstalk between cells of the innate immune system that is necessary for maximizing the efficiency of apoptotic cell removal.

## Introduction

The thymus provides a specialized microenvironment for T-lymphopoiesis. Its primary function is the generation of a T cell repertoire that ensures efficient immune responses to foreign substances, but precludes autoimmunity. Thymic selection occurs in multiple steps within distinct thymic microenvironments, where the interactions of developing thymocytes with thymic stromal cells are indispensable. Small numbers of thymic-resident macrophages, found mainly in the cortex, are believed to be responsible for the clearance of the millions of apoptotic cells that result from either failed positive selection or, to a lesser extent, negative selection [1].

The macrophage lineage is intrinsically heterogeneous for both surface phenotypes and immunological activities, presumably due to the intricate specialization of tissue macrophages present in local environments [2]. For example, the existence of two distinct monocytic lineages have been identified based on the expression of distinct chemokine receptors; a short-lived inflammatory subset that homes to inflamed tissues and a resident subset, with a longer half-life, that homes to non-inflamed tissues [3]. Thymic macrophages likely fall into the long-lived tissue resident subset, but on a whole the origin, cellular differentiation and migratory properties of thymic macrophages remain mostly unexplored. Moreover, there is conflicting information about both the phenotype and function of these cells [1], [4], [5], [6], [7]. These discrepancies likely arise from the difficulty in analyzing rare cell populations and also the different experimental approaches used to indentify the cells.

In this study, we analyzed the phenotype of a series of thymic-resident innate immune cells and identified two different macrophage subpopulations. Our data show that the efficient clearance of dying cells is achieved by a concerted effort of the resident macrophages and specialized innate cells that are recruited to the sites of extensive apoptosis. We show that a collaboration of these cells during this process is necessary to maximize the efficiency of apoptotic cell removal.

## Results

### Identification of thymic resident macrophages

**Figure 1:**
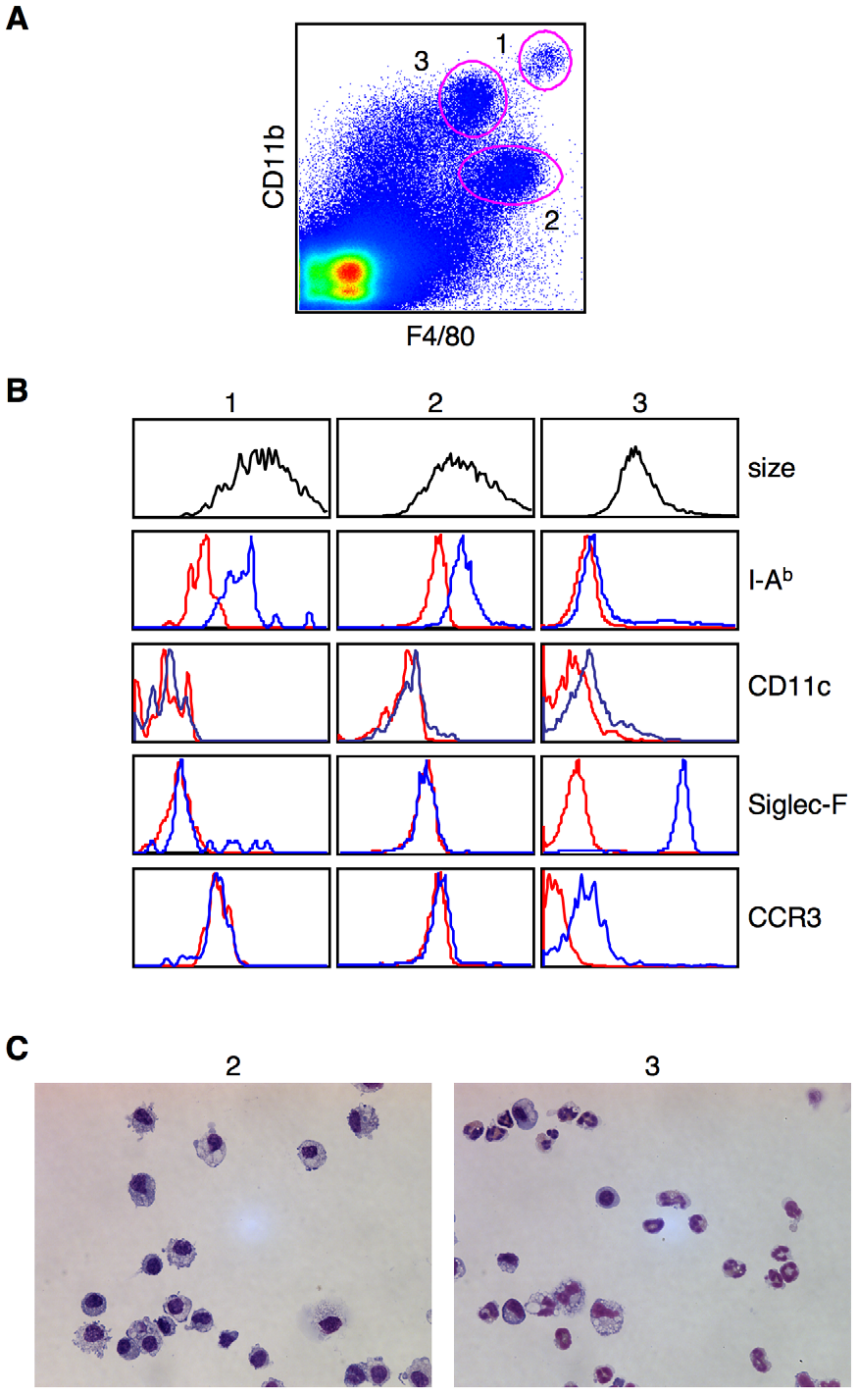
Heterogeneous phenotype of thymic resident myeloid cells. (**A**) Thymic stromal cells were enriched by collagenase/dispase digestion followed by percoll density gradient centrifugation. Cells were then stained with anti-CD11b and anti-F4/80 and analyzed by FACS. Red circles mark the three different cell populations discussed in the text. In this experiment, populations #1, #2 and #3 represented 0.02%, 0.43% and 0.61% of the analyzed cells, respectively. While the absolute cell number varied as a result of the preparation, the percentage of each cell type relative to each other was consistent over many experiments. (**B**) Size and surface marker expression (blue lines) of each population was determined by FACS. Populations were identified as in (**A**) and stained with the antibodies as indicated. Isotype controls (red lines) were used to correct differences in the autofluorescence of each cell population. (**C**) Individual cells from populations #2 and #3 were sorted onto glass slides followed by staining with H&E. Data are representative of more than ten experiments (a and b) and three experiments (c).

To directly study thymic resident macrophages, thymuses were digested with collagenase, followed by percoll gradient centrifigation to separate out the low-density cells, which we found were enriched for total thymic stromal cells. We tested a series of antibodies against various cell surface markers and found that a combination of anti-CD11b and anti-F4/80 was most effective for the examination of discrete populations of cells. By FACS, we identified three distinct cell subsets: (1) CD11b^hi^F4/80^hi^, (2) CD11b^lo^F4/80^hi^ and (3) CD11b^hi^F4/80^lo^ (Figure 1A). These cell subpopulations differed in their size, morphology and surface marker expression (Figure 1B).

Both the CD11b^hi^F4/80^hi^ (population #1) and CD11b^lo^F4/80^hi^ (population #2) cell subsets displayed a phenotype typical for macrophages. In particular, both populations consisted of large cells that were MHC-class II positive and CD11c negative (Figure 1B). In contrast to these two populations, the CD11b^hi^F4/80^lo^ cells (population #3) were smaller and MHC class II negative. Further analysis showed that the population #3 cells expressed both Siglec-F and CCR3. CCR3, the receptor for C-C type chemokines, is highly expressed by eosinophils and is important for their migration to sites of inflammation [8]. Siglec-F is the sialic acid-binding immunoglobulin-like lectin F, which appears to be a negative regulator of eosinophils activity [9]. Therefore, although these cells both express CD11b and F4/80 like thymic macrophages, they clearly are eosinophils. To further confirm the identity of populations #2 and #3, we sorted individual cells and stained with hematoxylin and eosin (H & E) (Figure 1C). The multi-lobed nucleus and presence of eosin positive cytoplasmic granules confirms the identity of the thymic resident eosinophils (population #3). The round nucleus, pseudopodia and what are, most likely, phagosomes, are all consistent with macrophage morphology (population #2).

Overall, our analyses identified two distinct thymic macrophage populations that can be distinguished by the differential expression of CD11b and F4/80. We also demonstrated that the thymus contains a sizable population of eosinophils, which were likely not excluded from previous studies of thymic macrophages.

### Innate immune cells are recruited into the cortex in response to enhanced apoptosis

**Figure 2:**
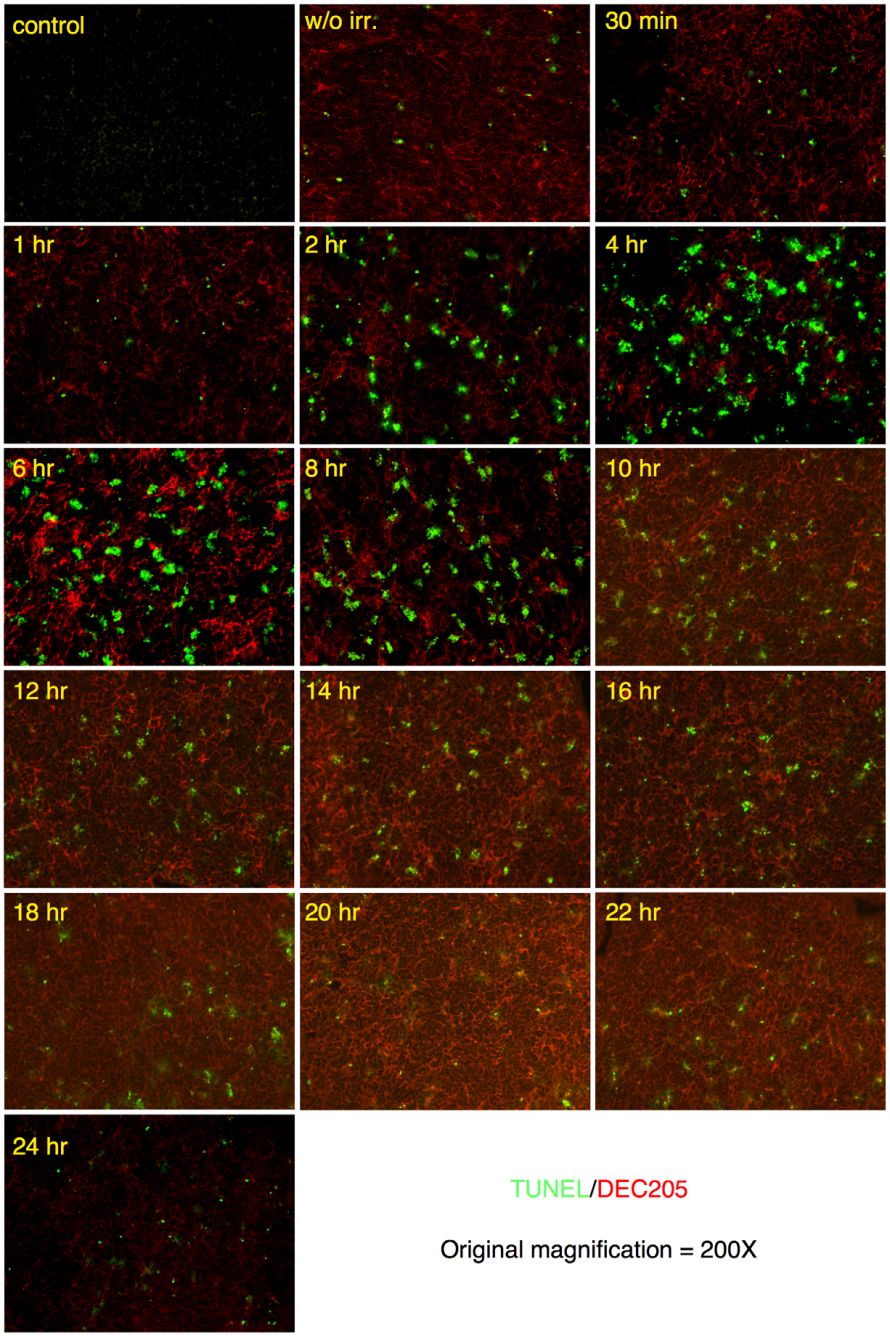
Time course analysis of apoptosis induced by irradiation. Thymuses from wild type B6 mice were harvested and frozen post-irradiation at the indicated times (1 hour through 24 hours). Sections were stained by TUNEL (green) to detect apoptotic cells and with anti-DEC205 (red) to mark the thymic epithelium. Panel labeled “control' was a section stained with secondary antibodies only. Panel labeled “w/o irr.” Was a section from a nonirradiated mouse. Original magnification was 200X.

**Figure 3:**
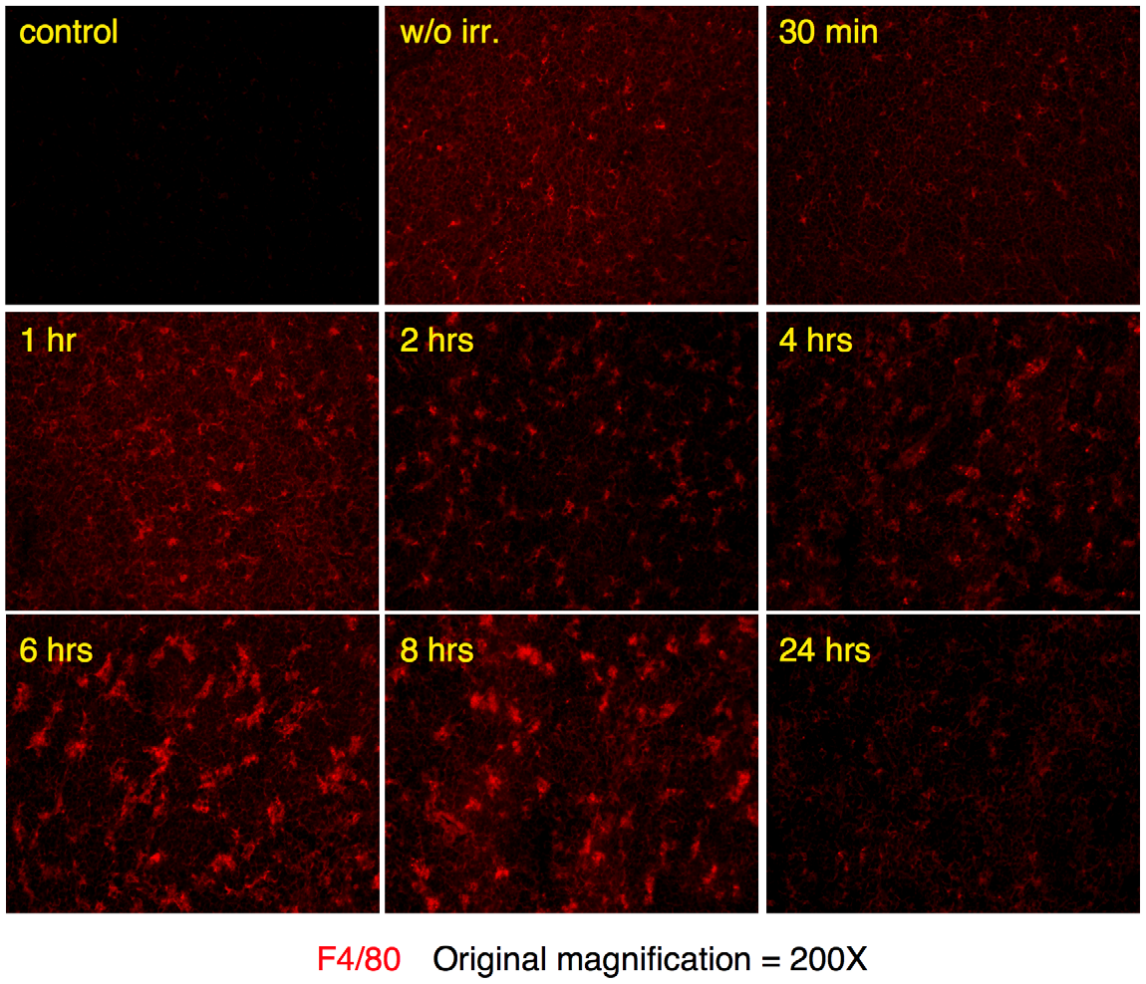
Time course analysis of F4/80 positive cells following irradiation. Thymuses from wild type B6 mice were harvested and frozen post-irradiation at the indicated times (1 hour through 24 hours). Sections were stained with anti-F4/80 (red), which identifies macrophages and eosinophils. Panel labeled “control' was a section stained with secondary antibody only. Panel labeled “w/o irr.” was a section from a nonirradiated mouse. Original magnification was 200X.

**Figure 4:**
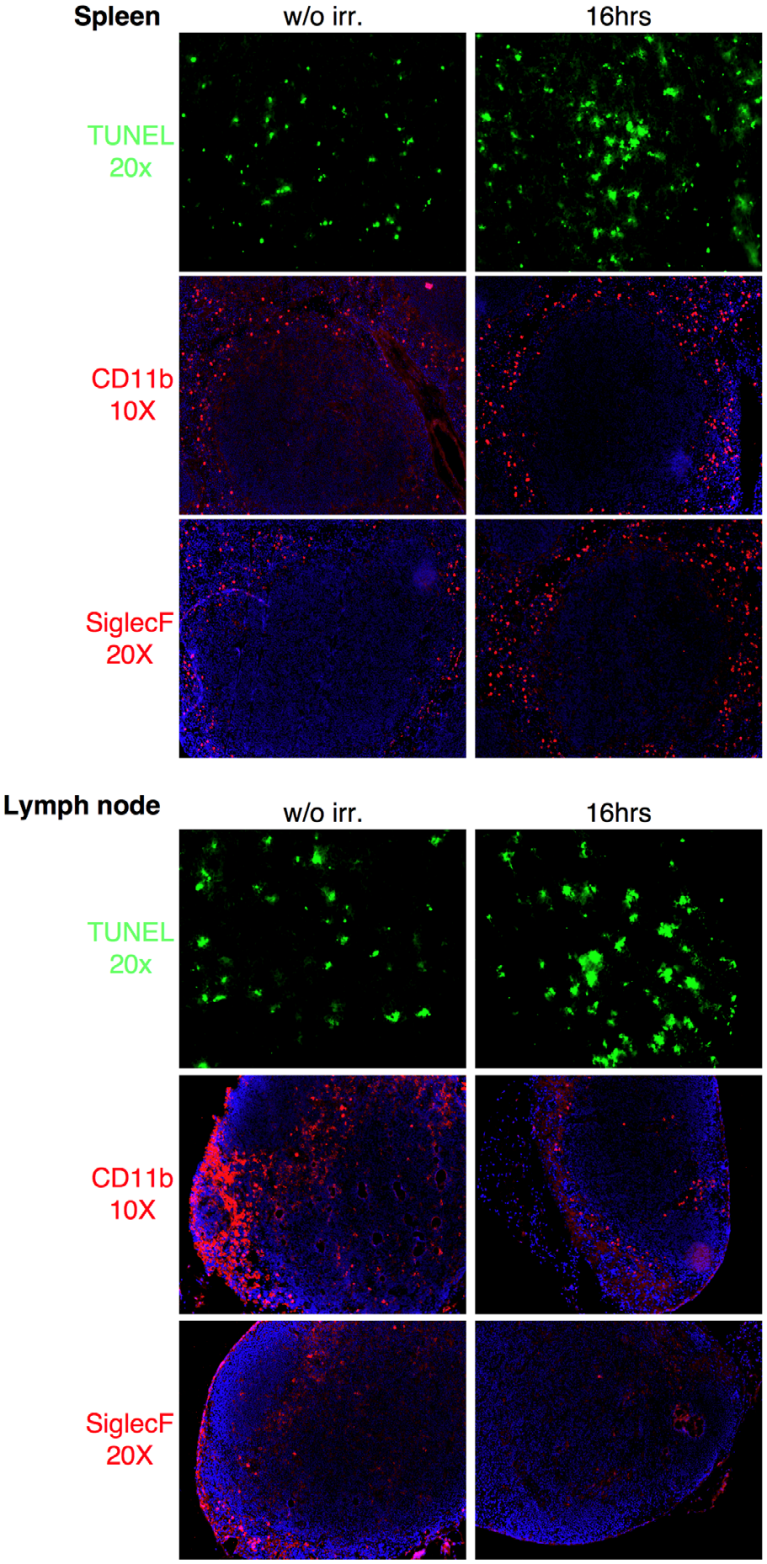
No increase in eosinophils or neutrophils in spleens or lymph nodes post-irradiation. Spleens and lymph nodes from wild type B6 mice were harvested and frozen either prior to irradiation or sixteen hours post-irradiation. Sections were stained by TUNEL to detect apoptotic cells, CD11b to detect neutrophils or SiglecF to detect eosinophils. Original magnification for each staining is listed in the figure. Data are representative of three independent experiments.

Cortical thymocytes undergo apoptosis when exposed to corticosteroids, irradiation or anti-CD3 antibodies [10], [11], [12]. In our experiments, synchronous apoptosis of thymocytes was induced by a single, low dose (1 Gy) of γ-radiation. This dose of radiation caused a rapid and highly reproducible increase in apoptotic cells in the cortex (DEC205^+^ area) [13], but had little impact on overall cellularity or structure of the thymus (data not shown). Time course analysis with TUNEL reagents showed that increased cell death could first be detected two hours after irradiation and peaked by four hours (Figure 2). Sixteen hours post radiation, TUNEL positive cells were greatly reduced and thymic homeostasis appeared to be completely restored within twenty-four hours (Figure 2). Nearly all apoptotic thymocytes appear as clusters. This “clustering” appears to be due to the localization of multiple TUNEL positive thymocytes, which based upon similarity in staining, are likely phagocytic F4/80^+^ cells (Figure 3). Finally, we analyzed spleens and lymph nodes from mice sixteen hours post irradiation (Fig. 4). The low levels of radiation used for our studies caused only a small increase in the frequency of apoptotic cells in these tissues. The tissues were also analyzed to determine if there was an increase in the frequency of neutrophils and/or eosinophils. As can be seen (Fig. 4) there was little or no increase for CD11b or SiglecF positive cells. Therefore, it is likely that the increase of eosinophils and neutrophils found in the thymus post-irradiation is due to the increased numbers of dying cells and not just an indirect consequence of irradiation.

**Figure 5:**
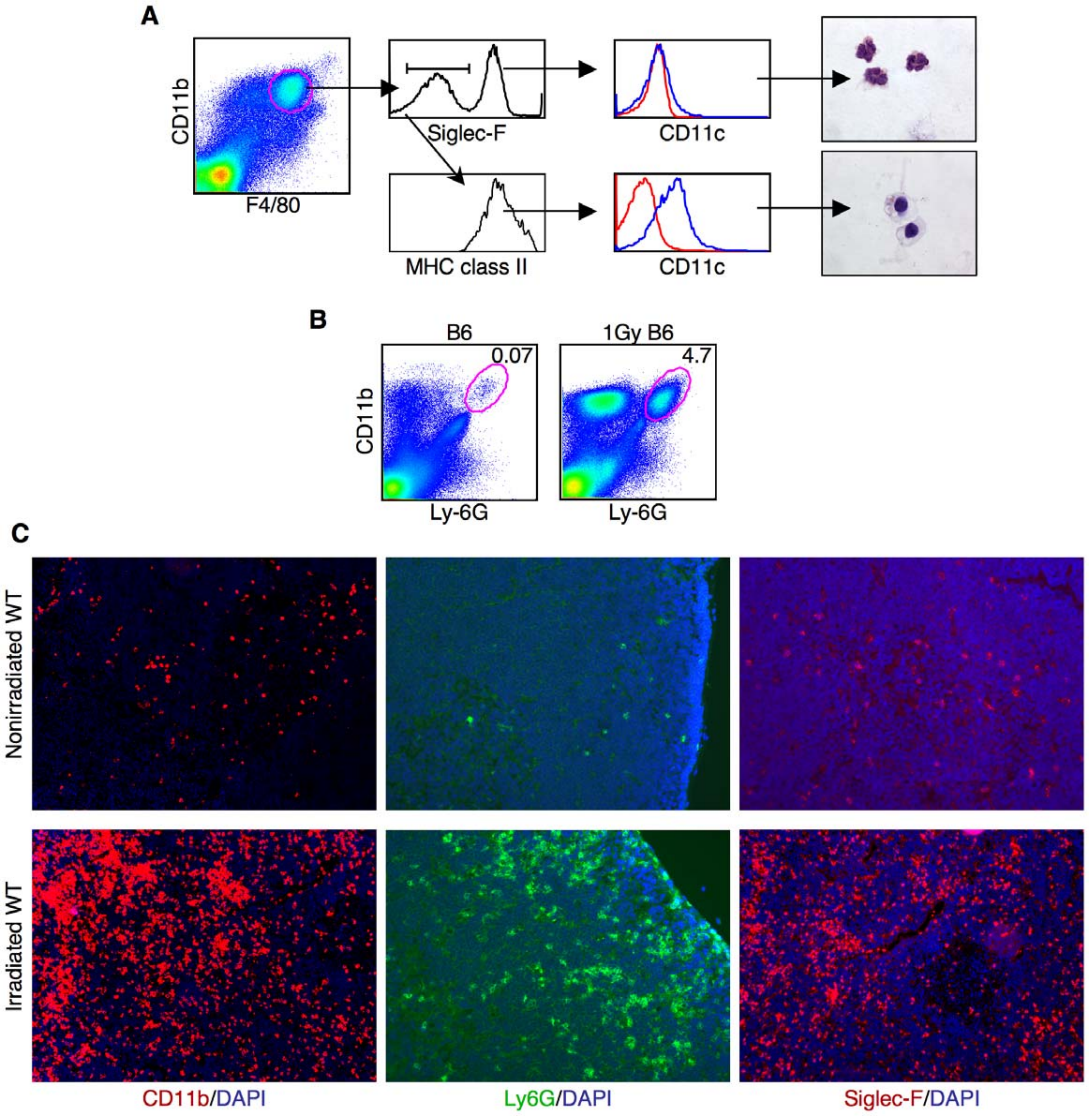
Migration of cells of the innate immune system into the thymic cortex following induction of thymocyte apoptosis. (**A)** FACS analysis of thymic stromal cells sixteen hours after irradiation collected as described in Figure 1. Microscopic analysis of sorted cells showed the morphological differences of eosinophils (top panel) and the myeloid type DCs (bottom panel). **(B)** Thymic stromal cells were stained for CD11b and Ly-6G before and after irradiation. The percentage of CD11b^+^Ly-6G^+^ cells is shown. **(C)** Thymuses were harvested from control (top panels) or irradiated (bottom panels) mice. Frozen sections were stained with CD11b^+^ (red), Ly-6G^+^ (green) and Siglec-F^+^ (red). Sections are representative of multiple samples from more than six experiments.

FACS analyses of thymic stromal cells sixteen hours after irradiation showed that substantial increase of CD11b^+^F4/80^+^ cells in the thymus of wild type mice (Figure 5A) as compared to nonirradiated mice (Figure 1A). Approximately half of these cells were eosinophils (Figure 5A). The Siglec-F negative fraction, on the other hand, expressed high levels of MHC class II and was CD11c^+^ (Figure 5A). The morphology of the MHCII expressing cells was also clearly distinct from the eosinophils (Figure 5A). The F4/80^+^, CD11b^+^, MHCII^+^, CD11c^+^ phenotype and the morphology together suggest that these are myeloid dendritic cells. Next, thymic stromal cells were stained with antibodies against Ly-6G and CD11b. Ly-6G (antibody clone 1A8) is expressed specifically by neutrophils and not by monocytes, eosinophils or lymphocytes [14], [15]. At steady state, CD11b^+^Ly-6G^+^ neutrophils represent less than 0.1% of the enriched stromal cell population, whereas after irradiation this increased to approximately 5% (Figure 5B). BrdU labeling studies showed that the increase in thymic stromal cells was not due to proliferation during the clearance of apoptotic cells (data not shown). Therefore, the increase of CD11b^+^F4/80^lo^ cells was most likely due to the migration of cells into the thymus in response to elevated apoptosis.

Next, thymic stromal cell populations were analyzed by immunohistochemistry before and after induction of apoptosis. The tremendous increase of CD11b^+^ cells post-irradiation demonstrated by FACS (Figure 5a, left panel) is easily visualized by immunohistochemistry (Figure 5c). The increase in total numbers of cells coupled with their large size makes the CD11b staining in portions of the cortex of the thymus appear to be nearly confluent. The CD11b positive cells consisted both of neutrophils (Figure 5c, middle panel) and eosinophils (Figure 5c, right panel). The infiltration was limited to the cortex, presumably because the more mature thymocytes in the medulla were not sensitive to the low dose of radiation that was used and, therefore, did not undergo apoptosis (data not shown).

**Figure 6:**
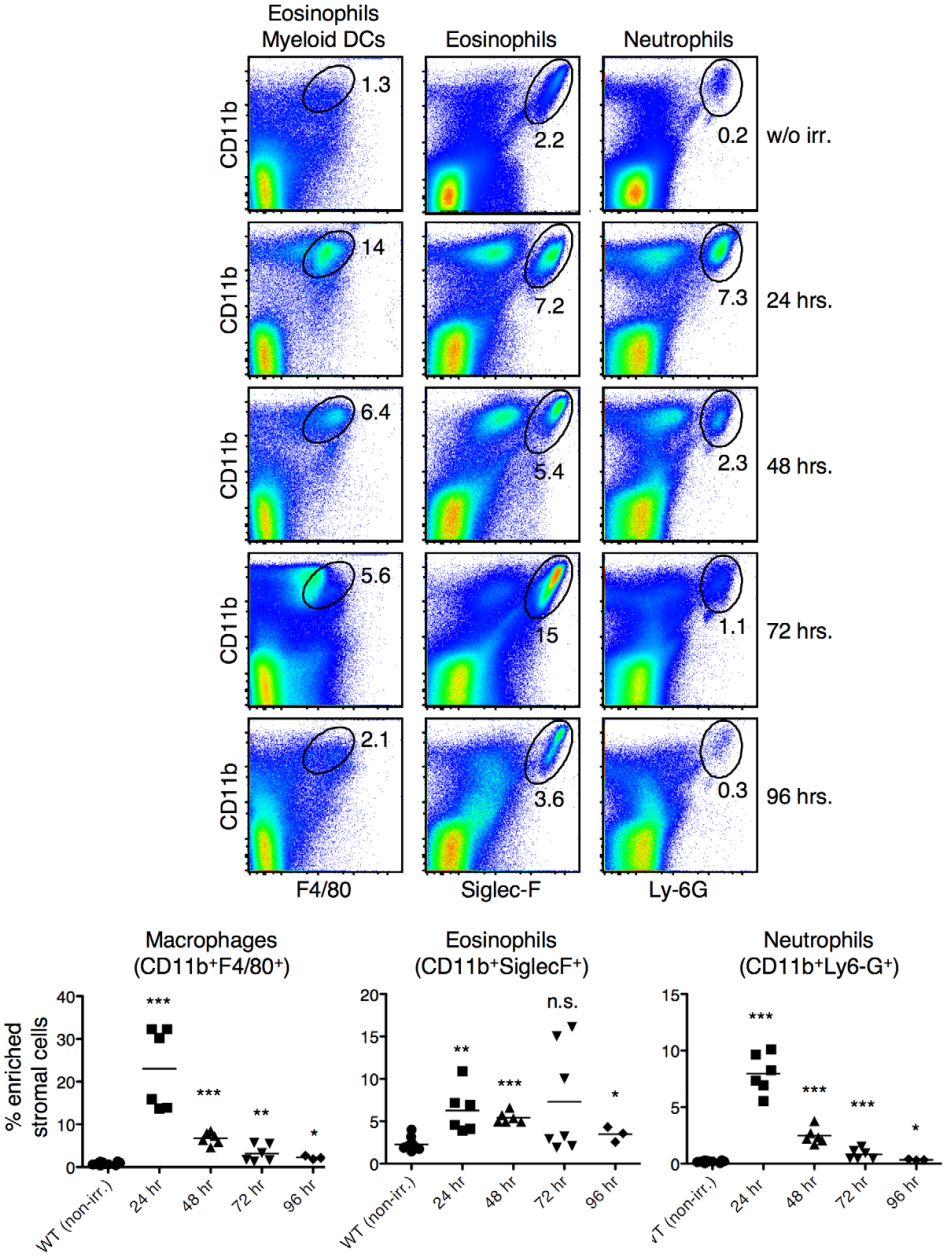
Influx of innate cells rapidly declines twenty-four hours after irradiation. Thymuses were harvested from mice at the indicated times after irradiation. Stromal cells were enriched as described and stained with anti-CD11b, -F4/80, SiglecF and -Ly-6G. Numbers in the plots are the percentage of cells within the electronic gate. N = 6 and the data show the results from two independent experiments. The mean is shown by a horizontal bar and statistics were calculated with 2-tailed, nonpaired Mann-Whitney test. *  =  <0.01; **  =  <0.001; ***  =  <0.0001.

By immunohistochemistry thymic homeostasis appeared to be restored by 24 hours post-irradiation. To more carefully analyze this, we harvested thymuses one, two, three and four days post irradiation and analyzed the cellular makeup by FACS (Fig. 6). The frequency of macrophages (CD11b^+^F4/80^+^), eosinophils (CD11b^+^SiglecF^+^) and neutrophils (CD11b^+^Lyg-G^+^) was clearly still increased 24 hours post-irradiation. Interestingly, a small, but statistically significant increase, in these populations was found even four days post irradiation, as compared to non-irradiated mice (Fig. 6). Overall, these data show that acute cell death in the thymus correlates with the infiltration of large numbers of neutrophils, eosinophils and myeloid dendritic cells.

### CD68 expression marks phagocytes responsible for clearing apoptotic cells

**Figure 7:**
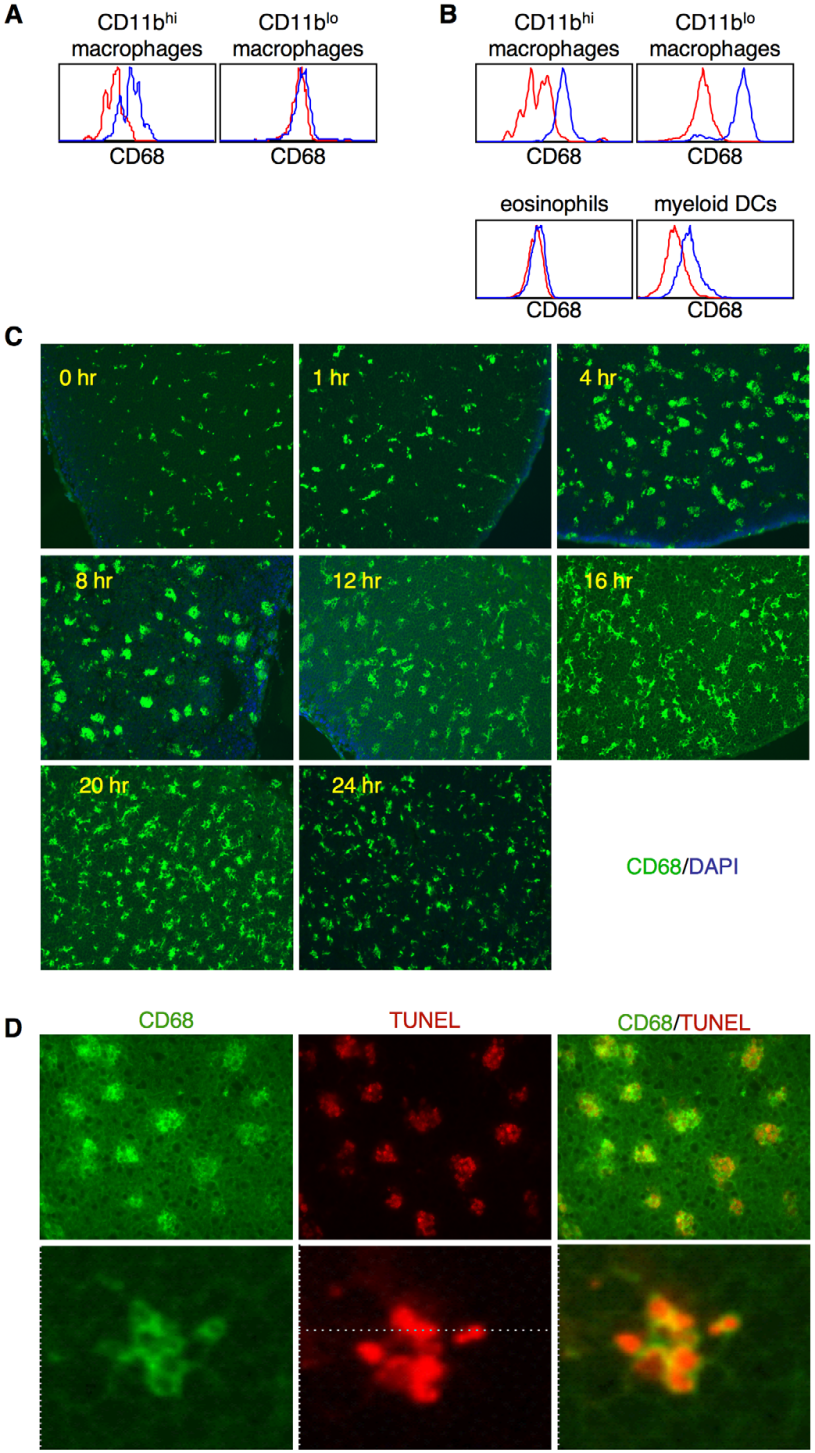
Nearly all apoptotic cells appear to be associated with CD68^+^ cells. (**A**) Thymic macrophages (CD11b^hi^ and CD11b^lo^) from non-irradiated mice were stained for surface CD68 expression (blue line). Red line is isotype control staining. (**B**) Intracellular staining for CD68 (blue line) in the indicated cell populations 16 hours post irradiation. Red line is isotype control staining. (**C**) Frozen sections of thymuses harvested 16 hours post irradiation were stained with DAPI and an antibody against CD68. Only thymic cortical areas are shown, since CD68^+^ cells were mainly found in the cortex (original magnification: 100X). (**D**) Clearance of apoptotic cells (top panels) was analyzed by staining thymic sections by TUNEL (red) and CD68 (green). Original magnification: 400X. Enlargement of a single CD68^+^ cell (bottom panels) shows multiple engulfed thymocytes, each ringed with CD68 (original magnification: 1000X). All sections are representative of multiple samples from more than six experiments.

The variety of cells that we found to be recruited to the thymus in response to cell death raised the question of which of the cells were actually involved in phagocytosis. Although CD68 (macrosialin) has sequence homology to the lysosomal/endosomal-associated membrane glycoprotein (LAMP) family, it has been shown to be transiently expressed on the cell surface of activated macrophages. Furthermore, it appears to play a role in phagocytosis [16]. It was interesting to find, therefore, that CD68 was constitutively expressed on the cell surface of the minor population #1, F4/80^hi^CD11b^hi^ macrophages prior to irradiation (Figure 7a). This suggested that these cells might be continuously involved in phagocytosis. Intracellular staining revealed that CD68 was also expressed at high levels by the population #2 F4/80^hi^CD11b^lo^ macrophages, the myeloid dendritic cells (MHCII^+^CD11c^+^ cells), but not by eosinophils (population #3) or neutrophils (Figure 7b and data not shown).

Thymic sections taken from mice 16 hours following irradiation were stained to determine if CD68 expressing cells were directly involved with clearance of apoptotic thymocytes. As can be seen in Figure 7c, nearly all CD68^+^ cells in the thymus became hyperextended and greatly enlarged. This change in shape and size closely correlated with the onset of extensive thymocyte apoptosis (Figure 2). Curiously, the altered morphology of the CD68^+^ cells persisted long after the TUNEL positive cells were cleared suggesting an extended, close interaction with other thymic stromal cells (Figure 7c).

Double staining with TUNEL and an antibody against CD68 (Figure 7d) showed that the CD68^+^ cells were the major cell type involved in phagocytosis of apoptotic thymocytes. Additionally, both types of thymic macrophages as well as the myeloid dendritic cells appeared to be involved in phagocytosis, since nearly all the CD68 cells visualized by immunohistochemistry also contained TUNEL positive cells. Finally, consistent with the expression of CD68 on lysosomal membranes, each apoptotic cell was circled with CD68 staining. This staining pattern strongly indicated that the TUNEL^+^ cells had been engulfed into the CD68^+^ cells rather than simply being attached to the surface of the CD68^+^ cells. The double staining with anti-CD68 and TUNEL also showed that each section of the CD68^+^ cells that was examined had multiple individual phagocytosed cells suggesting that, overall, each phagocytic cell likely contains many apoptotic thymocytes (Figure 7d). These data suggest that, in addition to the resident macrophages, additional cell types can be recruited to a site of acute cell death and participate in clearing cell corpses.

### Impaired clearance of apoptotic thymocytes in *Csf1* ^op/op^ mice

**Figure 8:**
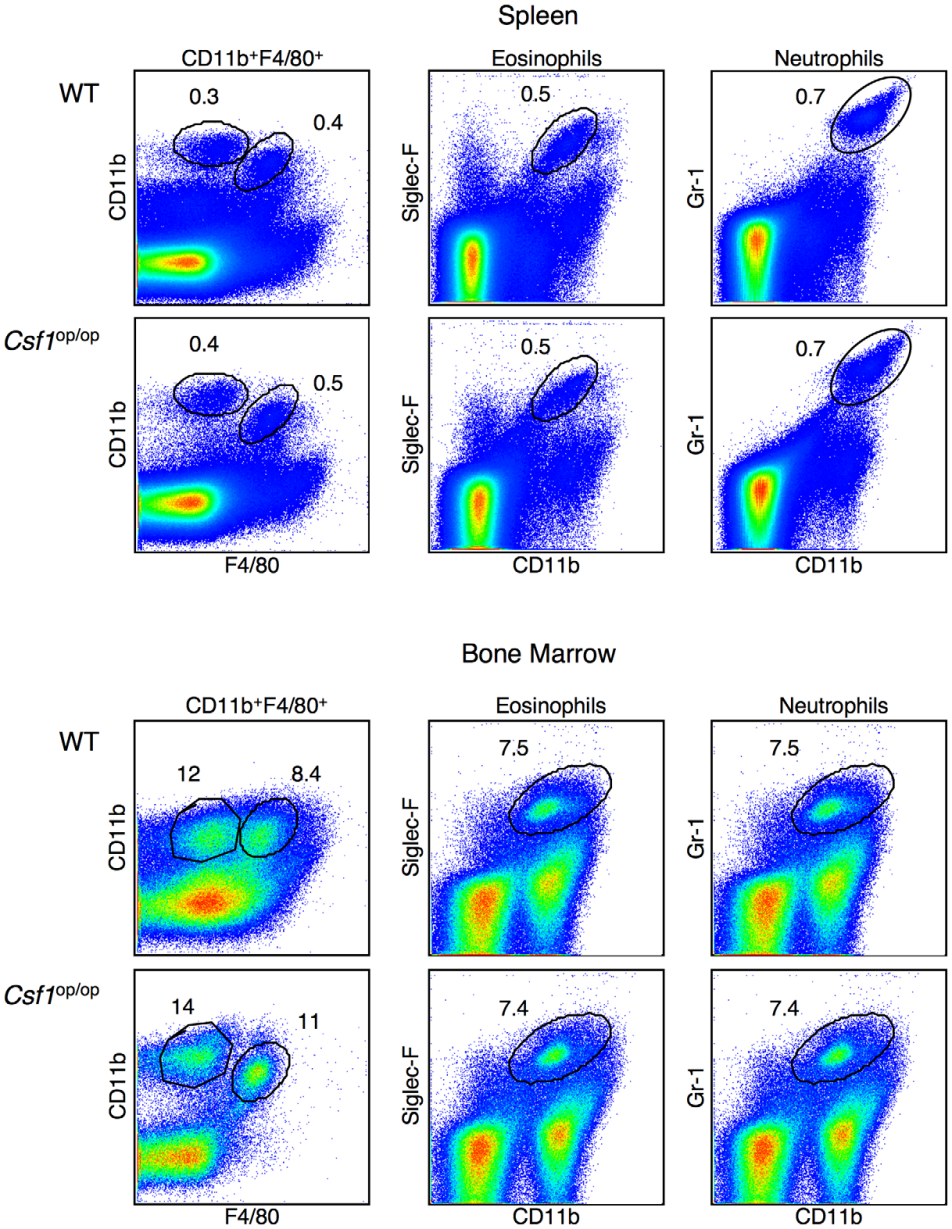
FACS analyses of the innate cells in the spleen and bone marrow from WT and *Csf ^op/op^* mice. Cells from the spleen and bone marrow from WT and *Csf^op/op^* mice were stained for CD11b and F4/80. Eosinophils and neutrophils were identified by staining cells for CD11b and SiglecF or CD11b and Gr-1, respectively. Percent of cells in each area is indicated in the Figure.

**Figure 9:**
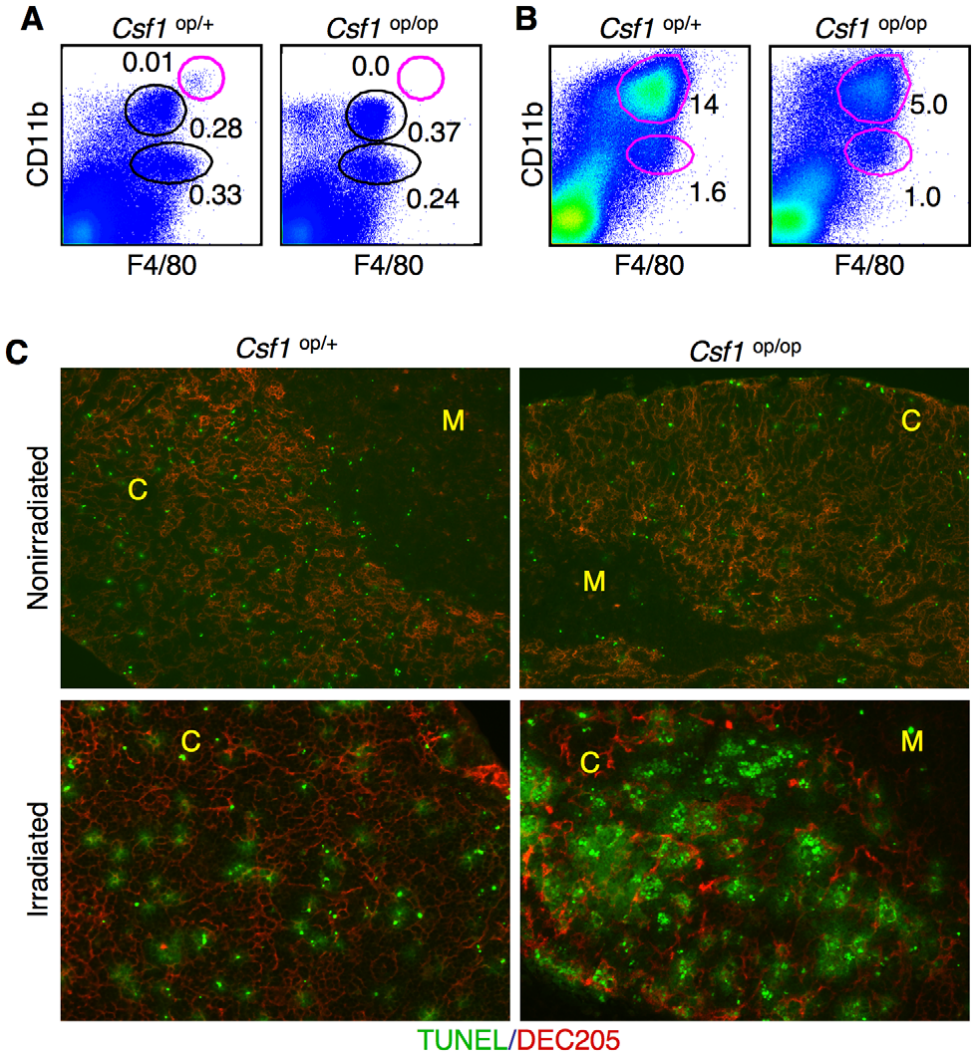
Impaired clearance of apoptotic thymocytes in *Csf1* ^op/op^ mice. (**A**) Thymic stromal cell subpopulations from WT (*Csf1* ^op/+^) and osteopetrosis (*Csf1* ^op/op^) mice were stained with the indicated antibodies and analyzed by FACS. Numbers indicate the percentage of cells within the circles. The red circle marks population #1, which is not present in *Csf1* ^op/op^ mice. (**B**) Comparison of thymic stromal cell subpopulations between WT (*Csf1* ^op/+^) and osteopetrosis (*Csf1* ^op/op^) mice 16 hours after irradiation. Thymic stromal cells were enriched and stained for CD11b and F4/80. (**C**) Representative images showing the frequency of apoptotic cells in WT (*Csf1* ^op/+^) and *Csf1* ^op/op^ mice in nonirradiated mice in mice 24 hrs receiving 1Gy irradiation. Apoptotic cells were detected by TUNEL (green) and their cortical localization was visualized by counterstaining of thymic sections with an antibody against DEC205 (red). Original magnification was 100X. Sections are representative of multiple samples from more than ten experiments.

Osteopetrosis (*Csf1* ^op/op^) mice have a defect in the expression of macrophage colony stimulating factor (*Csf1*) [17], [18], which causes a severe deficiency of mature macrophages. These mice, however, have normal numbers of mDCs, neutrophils and eosinophils in the spleen and bone marrow (Figure 8). We analyzed whether the development of thymic resident macrophages was also impaired in *Csf1* ^op/op^ mice. As shown in Figure 9a, there is little change in the percentage of the major thymic macrophage population (population #2; CD11b^lo^F4/80^hi^) in the absence of *Csf1*, however, the minor population of CD11b^hi^F4/80^hi^ (population #1) macrophages were completely missing. The *Csf1* ^op/op^ mice, therefore, presented us with an opportunity to examine apoptotic cell clearance in the thymus in the absence of one of the two macrophage populations we identified.

*Csf1* ^op/op^ mice and heterozygous (*Csf1* ^op/wt^) littermate mice were irradiated with 1 Gy of γ-radiation. Thymic stromal cells were harvested 16-hours after treatment and examined by FACS. Remarkably, infiltration of CD11b^+^ cells in the *Csf1* ^op/op^ mice was less than half of that found in the wild type littermates (Figure 9b).

Apoptotic T cells were detected at similar frequencies in both *Csf1* ^op/op^ and *Csf1* ^op/+^ thymuses at steady state. However, when apoptosis was induced by γ-irradiation, the removal of apoptotic cells in *Csf1* ^op/op^ mice was severely impaired as is evident by the frequency of TUNEL positive cells not associated with macrophages and also by the appearance of apoptotic cell “aggregates” (Figure 9c). These clusters of cells, which in some cases appeared to be cell “debris”, which apparently had not been ingested by macrophages, were not seen in wild type mice. Direct quantification of the frequency of apoptotic cells was not possible due to the near confluence of the TUNEL staining and the distribution of the cells within and around macrophages. Therefore, we examined multiple tissue sections across more than ten experiments to confirm reproducibility. Even twenty-four hours post-irradiation, TUNEL positive cells were still evident throughout the thymus in the mice missing the small, Csf1 dependent macrophage population (Figure 9c). These data suggest that failure to recruit CD11b^+^ cells to sites of acute cell death may decrease the efficiency of cell corpse clearance. It is possible, however, that although the frequency and phenotype of mDCs, neutrophils and eosinophils appeared normal in *Csf1* ^op/op^ mice, there was some unknown, underlying deficiency that affected the efficiency of phagocytosis in the thymus.

### Collaboration of innate immune cells is required for rapid clearance of apoptotic cells and tissue integrity

**Figure 10:**
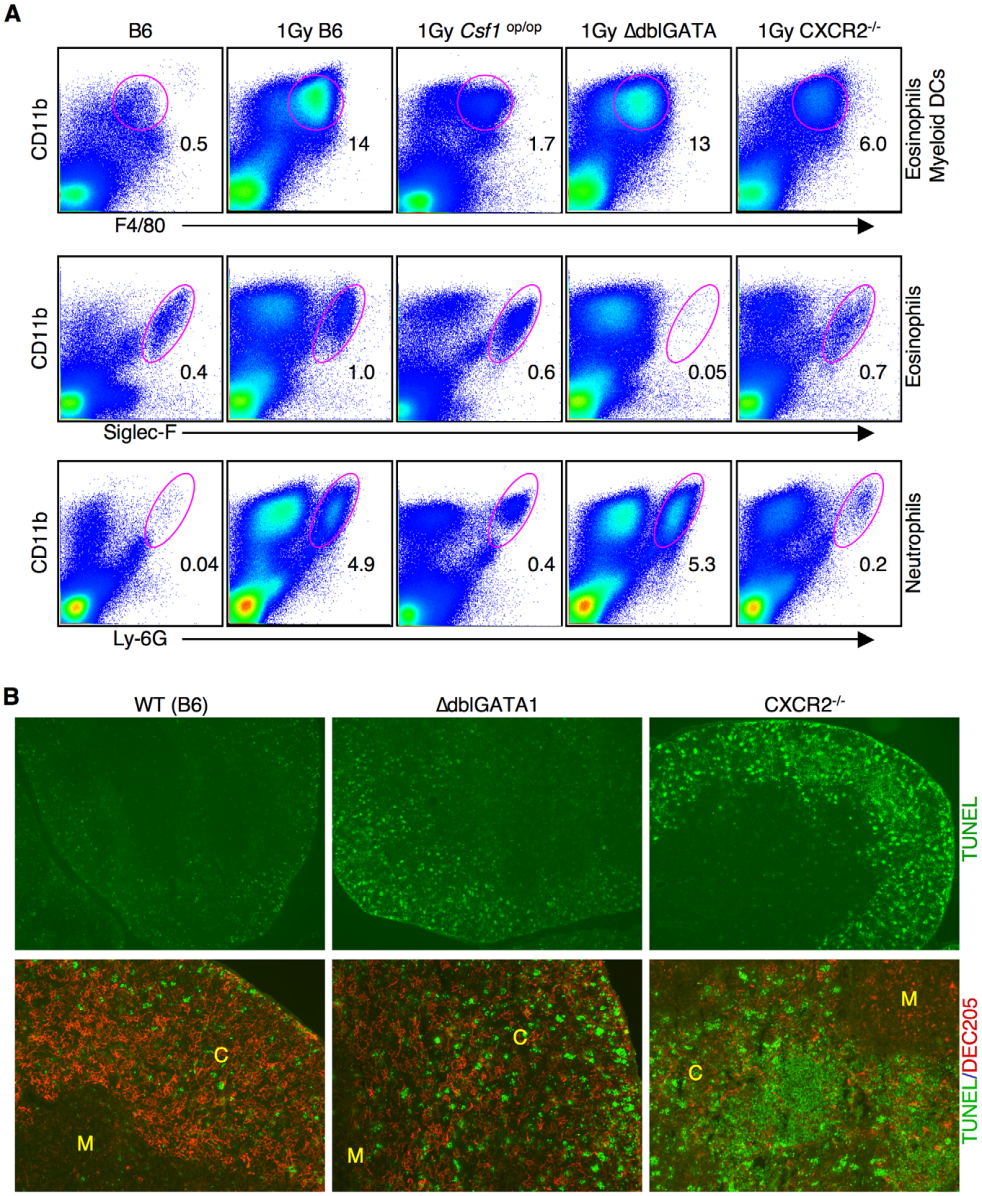
Eosinophils and neutrophils are essential for efficient clearance of apoptotic thymocytes. (**A**) FACS analyses of thymic stromal cells from wild type (C57Bl/6) mice, CSF1 deficient (*CSF1^op/op^*) mice, eosinophil deficient mice (ΔdblGATA), mice deficient for neutrophil migration (*CXCR2^−/−^*) 16 hours after apoptosis induction. Cells were stained with the indicated antibodies and analyzed for FACS. The percentage of CD11b^+^F4/80^lo^ (eosinophils and myeloid DCs) (top), eosinophils (middle) and neutrophils (bottom) is shown. (**B**) Frozen thymic sections were prepared 16 hrs after irradiation from wild type (B6), ΔdblGATA and CXCR2^−/−^ mice were stained either by TUNEL (green) or TUNEL and with an antibody against DEC205 (red). Top panels are at low magnification (original magnification: 50X) to show an “overview” of TUNEL positive cells in the thymus. Bottom panels (original magnification: 100X) shows apoptotic cells (TUNEL, green) and thymic cortical epithelial cells (DEC205^+^, red). Note the formation of cell clusters composed of non-engulfed apoptotic cells in CXCR2^−/−^ mice.

*Csf1* ^op/op^ mice had reduced migration of the CD11b^+^ cells in response to acute cell death in the cortex of the thymus. Additional FACS analysis showed that the infiltration of myeloid dendritic cells, neutrophils and, to a lesser extent, eosinophils was reduced as compared to wild type mice (Figure 10a). To individually study the roles that these cells might play in the clearance of apoptotic cells, we obtained mice in which eosinophils do not develop and mice in which neutrophil trafficking is nearly completely blocked. Mice specifically lacking myeloid dendritic cells to our knowledge are not available.

Eosinophils are specifically deficient in ΔdblGATA mice due to a deletion of a high affinity GATA-binding site in the GATA-1 promoter [19]. As expected, at steady state, ΔdblGATA mice do not have thymic eosinophils, but the number of thymic macrophages was equivalent to WT mice (data not shown). Therefore these mice were used to determine if eosinophils play a role in the clearance of apoptotic cells in the thymus. In response to induced apoptosis, migration of neutrophils and myeloid dendritic cells into the thymus was not affected in ΔdblGATA mice as compared to wild type mice (Figure 10a). Despite the normal number of macrophages and the recruitment of neutrophils and myeloid dendritic cells, TUNEL positive cells that had not been phagocytosed, as well as apoptotic aggregates, were greatly increased (Figure 10b, middle). The nature of the TUNEL staining, the cell aggregates and the distribution of apoptotic cells within or around the macrophages made direct quantification of the frequency of non-phagocytosed cells impossible. Results were, therefore, confirmed by examining multiple tissue sections from five independent experiments.

Next, the role of neutrophils in the clearance of apoptotic thymocytes was evaluated by using mice deficient for the chemokine receptor CXCR2 (CXCR2^−/−^ mice). These mice have a major defect in neutrophil migration to sites of inflammation [20]. CXCR2^−/−^ mice have normal numbers of thymic eosinophils and macrophages relative to WT mice at steady state (data not shown). The migration of neutrophils into the thymus 16 hours after irradiation was dramatically reduced (Figure 10a). Clearance of apoptotic T cells in the thymus of CXCR2^−/−^ mice was severely impaired (Figure 10b). Once again, TUNEL positive cells were also found either as free apoptotic cells or as large clusters of apoptotic cell “debris” (Figure 10b, bottom right panel). There also was a slight reduction in the recruitment of mDCs into the thymus of CXCR2^−/−^ mice (Figure 10a and data not shown). We cannot exclude the possibility that this dramatic failure of apoptotic cell clearance in CXCR2^−/−^ mice is resulted from the combined effects of the lack of neutrophil migration and somewhat reduced recruitment of mDC. However, the accumulation of apoptotic cells in CXCR2^−/−^ mice was more prominent than in *CSF1* ^op/op^ mice (Figure 9c), even though *CSF1* ^op/op^ mice had fewer myeloid DCs post irradiation (Figure 9b). These results suggest that neutrophils play a critical support role for the rapid recognition and the efficient removal of apoptotic cells by phagocytes.

Taken together, these data suggest that interactions among several cell types are necessary for the efficient removal of apoptotic cells. In particular, if the migration of eosinophils or neutrophils is prevented, the ability of professional phagocytes to clear dead cells is severely compromised.

## Discussion

Here we describe two distinct macrophage populations in the thymus. The numerically more prominent population was identified as being to be CD11^lo^F4/80^hi^ and expressed moderate levels of MHC class II. The second, relatively minor population had a CD11b^hi^F4/80^hi^ phenotype and also expressed moderate levels of MHCII. Interestingly, the CD11b^hi^F4/80^hi^ macrophages were completely missing in CSF-1 deficient mice. These CSF-1 dependent macrophages were also distinguished by being the only cells in the thymus to express CD68 at the cell surface. The lysosome-associated CD68 protein is typically only expressed at the cell surface of phagocytically active cells, suggesting that this minor population may be actively clearing cell carcasses that are a consequence of normal thymopoiesis.

CSF-1^op/op^ mice did not show an obvious thymic defect. Induction of synchronized apoptosis of cortical thymocytes by γ-irradiation of mice, however, revealed a marked decrease in apoptotic cell clearance. In particular, free TUNEL positive thymocytes and TUNEL positive “aggregates” were observed. This was in sharp contrast to wild type mice, in which TUNEL positive thymocytes were rapidly engulfed by CD68^+^ cells. The difference in the ability to clear apoptotic cells correlated with a reduced infiltration of eosinophils, neutrophils and myeloid dendritic cells to the thymus. Eosinophils have been shown to be recruited into the thymus in a TCR transgenic model of negative selection [21], [22], although the direct contribution of this cell type to the apoptotic cell clearance has not been studied [21].

Mice with specific defects in either eosinophil development or in neutrophil migration were used to understand the role that these cells were playing in the clearance of apoptotic cells in this acute situation. Remarkably, the loss of either the eosinophils or the neutrophils in the thymus was shown to have a dramatic impact on the efficiency of cell corpse clearance. Although there was a clear role for neutrophils and eosinophils for the clearance of cell corpses in the thymus during acute cell death, we did not observe quantifiable differences in cell clearance at steady state. These data are in agreement with a previous report, which shows that eosinophils can help macrophages in the detection of apoptotic cells and their subsequent removal [23]. Eosinophils are multifunctional leukocytes implicated in the pathogenesis of numerous inflammatory processes, including parasitic helminth infections and allergic diseases [24], [25]. Neutrophils are generally thought to be an indicator of inflammation. However, emerging evidence supports the view that eosinophils and neutrophils are also involved in homeostasis, tissue repair and enhancement of phagocytosis [26], [27]. A recent study has shown that Mincle, a molecule expressed by macrophages in response to various stresses, may trigger the beneficial recruitment of neutrophils in response to the thymic tissue damage [28]. Indeed, injection of anti-Mincle antibody was shown to block neutrophils migration to the thymus. An earlier study showed that the cytokine MIP-2 produced by macrophages that phagocytose apoptotic cells induced the accumulation of neutrophils at the site of acute cell death [22].

We also show that expression of macrosialin (CD68) closely correlates with cells that are actively clearing apoptotic thymocytes. The CD68 positive cells included the CSF-1 dependent and independent thymic macrophages as well as the myeloid dendritic cells. Indeed, by histology, nearly all TUNEL positive cells were associated with CD68 expressing cells and nearly all the CD68 positive cells were engorged with dying thymocytes. Therefore, although a definitive function for CD68 remains to be determined, its expression is clearly correlated with phagocytically active cells. Eosinophils and neutrophils, however, did not express CD68 and, therefore, do not appear to be directly involved in ingesting apoptotic cells. Nevertheless, these cells are critical for clearance of dying cells. Their function, therefore, is likely to be due to modulation of the activity of the macrophages and mDCs by undetermined signals.

Many studies have shown that the detection and elimination of apoptotic cells by professional phagocytic cells does not cause inflammation or tissue damage. One view is that engulfment of apoptotic cells is a passive process that occurs prior to cell lysis and, thereby the release of inflammatory mediators and damaging factors is prevented [29]. Data indicates, however, that apoptotic cells and macrophages can be actively involved in the generation of immunosuppressive microenvironments [30]. For example, upon recognition and phagocytosis of apoptotic cells, macrophages are now known to actively modulate their genetic programs in a way that leads to the inhibition of pro-inflammatory cytokine production [31]. Furthermore, there is an enhancement of anti-inflammatory mediators such as TGF-β [32]. Cell death, therefore, can actively promote immune tolerance. It is possible that differential recruitment and/or crosstalk with cells such as eosinophils or neutrophils influences this process.

Our data suggest that crosstalk among macrophages, eosinophils, neutrophils and myeloid dendritic cells is essential for maximizing the efficiency of apoptotic cell clearance, at least in acute situations. While we have only examined the ingestion of apoptotic thymocytes, it can be speculated that the crosstalk between cells of the innate immune system might be similarly required for tissue homeostasis in certain conditions associated with significant amounts of cell death. For example, this finding might be of particular importance for understanding the complex interplay between tumors and the immune system [33], [34], [35].

## Materials and Methods

### Mice

C57BL/6 and BALB/c mice were purchased from The Jackson Laboratory. *Csf1* ^op/+^ mice were provided by Dr. J. Pollard (Albert Einstein College of Medicine) and *Csf1* ^op/op^ were generated at Memorial Sloan-Kettering's Research Animal Resource Center by breeding of *Csf1* ^op/+^ mice. ΔdblGATA mice were provided by Drs. S. Orkin and A. Humbles (Harvard Medical School, Boston). CXCR2^−/−^ mice were provided by Dr. E. Pamer (Memorial Sloan Kettering Cancer Center, New York). All experiments involving mice were approved by the Institutional Animal Care and Use Committee of Sloan-Kettering Institute (New York).

### Reagents and Antibodies

The following antibodies were purchased from BD Pharmingen: anti-CD11b -APC (M1/70), anti-Siglec-F-PE (E50-2440), anti-CCR3-Alexa Fluor 647 (83103), anti-CD86-PE (GL1), anti-Ly-6G-FITC (1A8), anti-I-A^b^-FITC (AF6-120.1), Streptavidin-PE, Streptavidin-PE-Cy7. Anti-F4/80-biotinylated (Cl:A3-1) antibody, anti-CD68- FITC (FA-11) were purchased from Serotec. Streptavidin-FITC was purchased from Caltag. ApopTag® Fluorescein Direct in Situ Apoptosis Detection Kit was purchased from Chemicon. DAPI was purchased from Molecular Probes. Anti-DEC205 antibody was produced by the Monoclonal Antibody Core Facility at the Memorial-Sloan Kettering Cancer Center.

### Purification of thymic stromal cells

Thymuses were cut into small pieces and digested with a mixture of Collagenase/Dispase solution (1 mg/ml, Roche) containing DNase I (50 µg/ml, Roche) for two incubations of 30 min at 37°C while stirring gently. The enzyme reaction was stopped by resuspending the cells with media containing 5% FCS. The enzyme-digested cells and the cells in the supernatant were pooled and separated on a discontinuous Percoll density gradient (ρ = 1.01, 1.03, 1.05, 1.07 and 1.10) by centrifugation at 3,500 g for 20 min at RT. Light-density cells were collected from the second and third interfaces, which contain the majority of the thymic stromal cells.

### Induction and detection of apoptosis

To induce apoptosis, mice were exposed to 1 Gy of gamma irradiation from a Cesium source and sacrificed at various times post exposure. In situ detection of apoptosis was performed by a standard TUNEL staining (ApoTag Apoptosis Detection Kit, Chemicon). In short, thymic tissue was sectioned in 6 µm slices that were dried for 1 hour. Tissue sections were fixed with 4% formaldehyde for 30 min at RT, and then repeatedly washed with PBS. To permeabilize cells, tissue sections were incubated with 0.1% Triton X-100 for 2 min on ice. After washing in PBS, tissue sections were incubated in equilibration buffer for 1 min. TdT enzyme dissolved in reaction buffer was mounted onto the sections and incubated for 1 hour at 37°C. The reaction was stopped by incubating the sections with Stop/Wash buffer. To visualize the cortex, counterstaining was performed by incubating sections with anti-DEC205-biotin antibodies followed by strepavidin-PE. Fluorescent images were taken with the Axio Imager program (Zeiss).

### Flow Cytometry

Cells were incubated first with Fc-Block (BD Pharmingen) and stained with the relevant antibodies for 30 min on ice. Multiparameter analyses were performed on Cyan Flowcytometer (Dako Cytomation) and data were analyzed with the FlowJo software (Tree Star). Dead cells were excluded by staining with DAPI and cell doublets were excluded by monitoring the pulse width.

### Immunohistochemistry and immunofluorescence

Thymuses were harvested, embedded in OCT compound (Sakura) and frozen on dry ice. Frozen sections were mounted on slides and fixed in acetone. Cells were labeled with antibodies for CD68, CD11b, Siglec-F, Ly-6G or DEC205. DAPI staining was performed subsequent to specific staining, to visualize the relative locations of the cortex and the medulla. Each experiment was carried out at least three times. Multiple frozen sections were examined for each experimental condition. Images used for all figures were highly representative of data collected from multiple experiments.
